# Tumor Burden Talks in Cancer Treatment with PEGylated Liposomal Drugs

**DOI:** 10.1371/journal.pone.0063078

**Published:** 2013-05-10

**Authors:** Yi-Yu Lin, Hao-Wen Kao, Jia-Je Li, Jeng-Jong Hwang, Yun-Long Tseng, Wuu-Jyh Lin, Ming-Hsien Lin, Gann Ting, Hsin-Ell Wang

**Affiliations:** 1 Biomedical Imaging and Radiological Sciences, National Yang-Ming University, Taipei, Taiwan; 2 Taiwan Liposome Company, Taipei, Taiwan; 3 Institute of Nuclear Energy Research, Taoyuan, Taiwan; 4 Taipei City Hospital Zhongxiao Branch, Taipei, Taiwan; 5 National Health Research Institutes, Miaoli, Taiwan; The University of Chicago, United States of America

## Abstract

**Purpose:**

PEGylated liposomes are important drug carriers that can passively target tumor by enhanced permeability and retention (EPR) effect in neoplasm lesions. This study demonstrated that tumor burden determines the tumor uptake, and also the tumor response, in cancer treatment with PEGylated liposomal drugs in a C26/tk-luc colon carcinoma-bearing mouse model.

**Methods:**

Empty PEGylated liposomes (NanoX) and those encapsulated with VNB (NanoVNB) were labeled with In-111 to obtain InNanoX and InVNBL in high labeling yield and radiochemical purity (all >90%). BALB/c mice bearing either small (58.4±8.0 mm^3^) or large (102.4±22.0 mm^3^) C26/tk-luc tumors in the right dorsal flank were intravenously administered with NanoVNB, InNanoX, InVNBL, or NanoX as a control, every 7 days for 3 times. The therapeutic efficacy was evaluated by body weight loss, tumor growth inhibition (using calipers and bioluminescence imaging) and survival fraction. The scintigraphic imaging of tumor mouse was performed during and after treatment.

**Results:**

The biodistribution study of InVNBL revealed a clear inverse correlation (*r* ^2^ = 0.9336) between the tumor uptake and the tumor mass ranged from 27.6 to 623.9 mg. All three liposomal drugs showed better therapeutic efficacy in small-tumor mice than in large-tumor mice. Tumor-bearing mice treated with InVNBL (a combination drug) showed the highest tumor growth inhibition rate and survival fraction compared to those treated with NanoVNB (chemodrug only) and InNanoX (radionuclide only). Specific tumor targeting and significantly increased tumor uptake after periodical treatment with InVNBL were evidenced by scintigraphic imaging, especially in mice bearing small tumors.

**Conclusion:**

The significant differences in the outcomes of cancer treatment and molecular imaging between animals bearing small and large tumors revealed that tumor burden is a critical and discriminative factor in cancer therapy using PEGylated liposomal drugs.

## Introduction

Conventional anticancer drugs exhibited many adverse effects resulting from systemic toxicity or non-specific retention in normal tissues. Lipid-based nanoparticles have been widely used as drug delivery agents for small molecule anticancer drugs to improve the pharmacokinetic profile and therapeutic efficacy [1]. Liposomes are self-assembling colloidal particles composed of lipid bilayer membranes that enclose a small volume of aqueous medium for drug encapsulation [2]. The development of surface modified liposomes in the last decade has rekindled interest in the clinical application for cancer treatment. Coating liposomes with hydrophilic polymers such as polyethylene glycol (PEG) gives them a form of steric barrier against interactions with plasma proteins, such as opsonins and lipoproteins [3]. Incorporation of PEG-derivatized lipids into the liposomes also inhibits liposome-induced complement activation [4] and escapes capture by the mononuclear phagocyte system [5]. Consequently, PEGylated liposomes remain in the circulation for prolonged periods, thereby conferring on entrapped agents the pharmacokinetic profile of the lipid carrier rather than that of free drugs [6].

PEGylated liposomes are capable of carrying drugs and providing passive targeting to tumors by enhanced permeability and retention (EPR) effect through the leaky vasculatures of tumor [1], [7]. However, some physiological barriers limited the delivery of macromolecules to tumor, such as heterogeneous blood flow, raised tumor fluid interstitial pressure and large transport distances in the tumor interstitium [8]. Beaney *et al.* showed that the tumor vascular volume increases at the early stages of tumor development, while decreasing as tumor grows up [9]. The necrotic areas become broadening and the average blood flow rate is decreased in a large tumor. The raised interstitial fluid pressure in a large tumor also reduced the transvascular delivery of macromolecules [10]. These effects might reduce extravasation of liposomes and result in poor therapeutic efficacy of a large tumor.

Vinorelbine (VNB) is a semi-synthetic vinca alkaloid (5′-nor-anhydro-vinblastine) that differs from others by a substitution of the catharantine moiety to the molecule [11]. The vinca alkaloids are known to inhibit cell proliferation through disruption of microtubules by reversible binding to tubulin, which results in mitotic spindle dissolution and metaphase arrest in dividing cells [12]. VNB has a favorable toxicity profile and activity against a wide range of human malignancies, including non-small cell lung cancer, breast cancer, ovarian cancer, and esophageal squamous cell carcinoma [13], [14], [15]. In-111, a radionuclide commonly used for scintigraphic imaging (t_1/2_ 2.81 days, 172 and 247 keV photons emission), can emit in average 14.7 Auger electrons per decay [16]. Auger electrons-emitting radionuclides are highly toxic to cells when internalized into the cytoplasm, especially if incorporated into DNA, may cause chromosome damage and have been suggested as antitumor agents [17], [18], [19], [20].

In this study, we aim to evaluate the influence of tumor size on the therapeutic efficacy after treated with ^111^In radionuclide and/or VNB chemodrug-encapsulated PEGylated liposomes in a C26/tk-luc colon carcinoma-bearing mouse model. The tumor uptake post intravenous administration of InVNBL in mice bearing various sizes of tumors was investigated. NanoX (PEGylated liposomes vehicle, as control) and three kinds of liposomal drugs, NanoVNB (NanoX encapsulated with VNB for chemotherapy), InNanoX (NanoX containing In-111 for radionuclide therapy) and InVNBL (NanoX carrying both VNB and In-111 for combination therapy), were employed to treat mice that bearing different sizes of C26/tk-luc colon carcinoma. The influence of tumor burden on tumor treatment with various liposomal drugs was examined. The scintigraphic imaging of mice after treated with InNanoX and InVNBL was also conducted during the period of treatment.

## Materials and Methods

### Ethics statement of animal work

The animal study was carried out in strict accordance with the recommendations in the Guide for the Care and Use of Laboratory Animals of the National Laboratory Animal Center. The protocol was approved by the Institutional Animal Care and Use Committee of National Yang-Ming University, Taiwan. (Permits Number: 980818 and 991208). The imaging studies were performed under 1–3% isoflurane anesthesia. All animals were sacrificed by carbon dioxide narcosis, and all efforts were made to minimize suffering.

### Materials

Cell culture materials were obtained from GIBCO BRL (Grand Island, NY, USA). Poly-Prep® chromatography column (40×8 mm) for gel purification was purchased from Bio-Rad (Hercules, CA, USA) and Sephadex™ G-50 Fine gel was purchased from Amersham Biosciences (Pittsburgh, PA, USA). 8-Hydroxyquinoline (oxine) was purchased from Sigma-Aldrich Corporation (St. Louis, MO, USA). No-carrier-added ^111^InCl_3_ (in 0.05 M HCl; 37–370 MBq) was obtained from Institute of Nuclear Energy Research (Taoyuan, Taiwan). All other chemicals were purchased from Merck (Whitehouse Station, NJ, USA).

### Cell line and tumor-bearing mouse model

The C26/tk-luc murine colon carcinoma stable cell line was a gift from Prof. Hwang. It was established and used as described in the previous publications [19], [21] and was maintained in RPMI 1640 supplemented with 10% fetal bovine serum. Male BALB/c mice (5- to 6-weeks-old) were obtained from National Laboratory Animal Center (Taipei, Taiwan). Mice were subcutaneously inoculated with 2×10^5^/100 µL C26/tk-luc cells in the right dorsal flank. The tumor size was estimated based on the formula: (length×width^2^)/2. Three mice were inoculated every 2 days to grow C26/tk-luc carcinoma with different size (*n* = 12; the tumor volume spans a range of 27.6 to 623.9 mm^3^) for evaluating the effect of tumor burden on InVNBL uptake. Other mice after inoculation for 12 and 14 days (tumor size were 58.4±8.0 mm^3^ and 102.4±22.0 mm^3^) were ready for use in therapeutic efficacy studies.

### Preparation of liposomal drugs NanoX, NanoVNB, InNanoX and InVNBL

The PEGylated liposomes, NanoX, were composed of distearoylphosphatidylcholine (DSPC), cholesterol, and polyethylene glycol-distearoylphosphatidylethanolamine (PEG-DSPE) (molar ratio, 3∶2∶0.045). NanoX was then encapsulated with VNB (350 µg VNB/µmol phospholipid) to produce NanoVNB. Both NanoX and NanoVNB were obtained from Taiwan Liposome Company (Taipei, Taiwan). NanoX and NanoVNB were labeled with ^111^In-oxine to afford InNanoX and InVNBL. The labeling method was detailed in our previous report [22]. Briefly, ^111^In-oxine was dissolved in 20 µL of ethanol and added with 80 µL of distilled water, and then incubated with 2 mL of NanoX/NanoVNB for 30 minutes at 37°C to afford InNanoX and InVNBL. The phospholipid concentration of all these liposomal drugs was 5.90 µmol/mL.

### Quality control of ^111^In-labeled liposomal drugs InNanoX and InVNBL

The entrapment of In-111 within NanoX/NanoVNB were assayed by loading about 100 µL of ^111^In-labeled product onto a column containing Sephadex™ G-50 Fine gel and eluting with normal saline. The labeling efficiency was determined by dividing the radioactivity in PEGylated liposomes fractions after separation with the total radioactivity before separation. The particle size of NanoX/NanoVNB and InNanoX/InVNBL was determined using a Zetasizer Nano ZS (Malvern, Worcestershire, UK).

### Effect of tumor burden on InVNBL uptake in biodistribution study

Mice with different tumor size (*n* = 12) were intravenously (i.v.) injected with 3.7 MBq/100 µL of InVNBL and then sacrificed at 48 h post injection by CO_2_ asphyxiation. The tumors were excised and weighed. The radioactivity of tumors was measured with a Wallac 1470 Wizard Gamma counter (GMI, Inc., Ramsey, Minnesota, USA). Data were expressed as percentage of injected dose per gram of tumor (%ID/g), and the relationship between InVNBL uptake and tumor burden was examined.

### Treatment protocol and therapeutic efficacy evaluation of liposomal drugs in tumor-bearing mice

The liposomal drugs treatment was initiated when the tumor burden of mice reached 58.4±8.0 mm^3^ (small-tumor group) and 102.4±22.0 mm^3^ (large-tumor group), respectively. All treatments were conducted at Day 0, 7 and 14 (total 3 doses) via intravenous injection. Four small-tumor groups (*n* = 6 for each group) and four large-tumor groups (*n* = 9 for each group) of mice were treated with NanoVNB (3 mg VNB/kg body weight), InNanoX (In-111, 37 MBq), InVNBL (3 mg VNB/kg body weight; In-111, 37 MBq) and NanoX (as control), respectively [18], [19]. The therapeutic efficacy was evaluated based on body weight loss, tumor growth inhibition (determined by using calipers and bioluminescence imaging, the phenomenon of photon flux of bioluminescence imaging in the developing tumors was related to the tumor size [19]) and survival fraction. The tumor size was measured thrice a week to document tumor growth. Tumor sizes were expressed as mean ± S.E.M.. The mean tumor growth inhibition rate (MGI) [19], [23], [24] was calculated according to the formula:
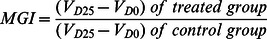
The enhancement in therapeutic efficacy after combined drugs treatment was evaluated by using the combination index (CI) [23], [25]: CI = expected tumor growth inhibition rate/observed mean tumor growth inhibition rate; expected tumor growth inhibition rate of combined drug treatment = mean tumor growth inhibition rate of drug A only×mean tumor growth inhibition rate of drug B only. In this study, drug A is NanoVNB and drug B is InNanoX. A combination index greater than 1 indicates a synergistic effect, while that smaller than 1 indicates less than an additive effect. The MGI and CI were determined at 25 days post initiation of treatment. The survival fraction of mice and the derived mean survival time (MST) and median survival time [26] were recorded. Mice were euthanized when the tumor burden was larger than 2500 mm^3^. The experiment was terminated 50 days after initiation of treatment.

### Bioluminescence imaging (BLI)

**Figure 1:**
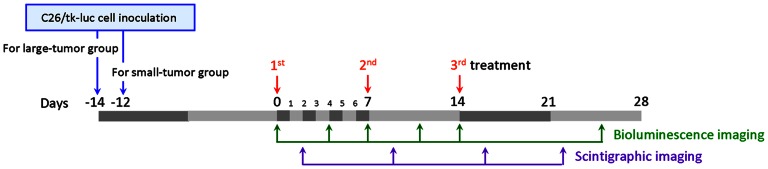
Schematic diagram of liposomal drugs dosing and imaging schedule. Mice were treated with liposomal drugs (NanoVNB, InNanoX, InVNBL and NanoX as a control) at Day 0, 7 and 14. Scintigraphic imaging of C26/tk-luc colon carcinoma-bearing mice treated with InVNBL was conducted on Day 2, 9, 16 and 22. Bioluminascence imaging was performed on Day 0, 4, 7, 11, 14 and 25.

The bioluminescence imaging of tumor-bearing mice was carried out at designated time points (Day 0, 4, 7, 11, 14 and 25, Fig. 1) post initiation of treatment. The mice were intraperitoneally injected with 150 mg/kg d-luciferin 15 min prior to image acquisition. The mice were anesthetized with 1–3% isoflurane (Abbott Laboratories, Queenborough, Kent, England) and were moved to a warmed stage in the chamber and continuous exposure with 1–3% isoflurane for sustained sedation during imaging. The photons emitted from the mice (positioned prone) *in vivo* were acquired for 1 min using an IVIS50 Imaging System (Xenogen, Alameda, USA). Regions of interests (ROIs) from displayed images were drawn around the tumor and quantified as photons/second (ph/s) using the Living Image software (Xenogen).

### Scintigraphic imaging

The scintigraphic imaging of tumor-bearing mice was conducted at 48 h after each treatment (Day 2, 9 and 16, Fig. 1) and 8 days post the third treatment. The mice treated with 37 MBq (1.0 mCi) of InVNBL/InNanoX through the tail vein were anesthetized with 1–3% isoflurane using a vaporizer system (A.M. Bickford, Wales Center, NY, USA). A dual head gamma camera (E. Cam Multiangle Cardiac, Siemens, Munich, Germany) equipped with a 4 mm pinhole collimator and an ICON P computer system (Siemens, Munich, Germany) was used for the scintigraphic imaging. The mice were placed prone on the bed. The images were acquired in a 256×256 matrix for 20 min, ROIs were drawn over the tumor area and the same region was copied to the contralateral muscle. The tumor-to-muscle ratios (T/M) were calculated on the basis of counts per pixel in the regions of interests.

### Statistical analysis

The student t-test was used for group comparisons. Values of *p*<0.05 were considered significant.

## Results

### Preparation and quality control of InNanoX and InVNBL

The labeling yield and the radiochemical purity of InNanoX and InVNBL were all greater than 90%. The radiochemical purity of InNanoX and InVNBL at 37°C after incubation in mouse plasma was 91.8±3.6% and 92.1±2.5% at 24 h, 85.6±4.2% and 84.8±5.1% at 72 h (*n* = 3), respectively, indicated a high *in vitro* stability of these liposomal drugs. The particle size of InNanoX and InVNBL was 98±5.6 nm and 100±5.3 nm after ^111^In-oxine labeling, respectively, similar to that of NanoX and NanoVNB (96±3.3 nm and 98±7.9 nm).

### Effect of tumor burden on InVNBL uptake in biodistribution study

**Figure 2:**
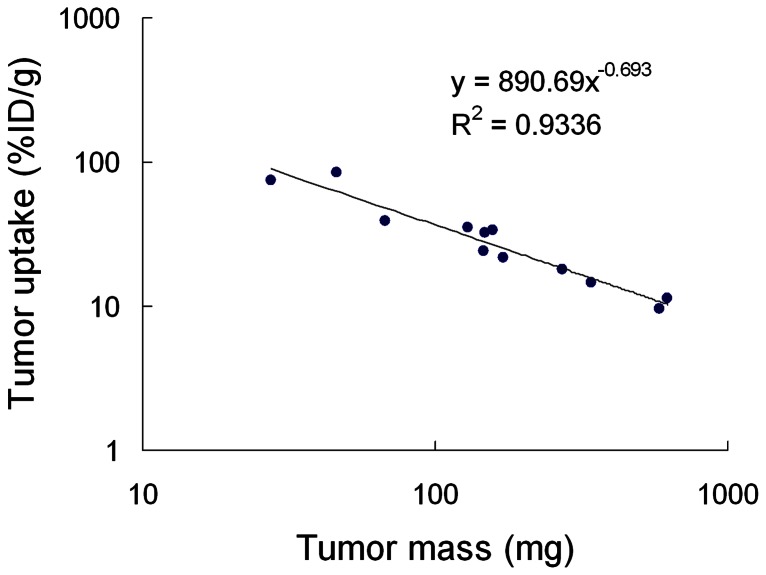
Relationship between tumor uptake (%ID/g) and tumor mass at 48 h after administration of InVNBL in mice bearing differently sized C26/tk-luc tumors.

There was a clear inverse correlation between the tumor uptake of InVNBL and the tumor mass, with a determined coefficient of *r* ^2^ = 0.9336 (Fig. 2). The tumor uptake of InVNBL decreased exponentially from 85 to 10%ID/g when the tumor mass increased from 30 to 650 mg. No significant difference in normal organs uptakes of InVNBL was observed between mice bearing differently sized tumors. Distribution of InVNBL in various tissues of C26/tk-luc tumor-bearing mice post intravenous injection (p.i.) has been detailed in our previous studies [19]. The tumor uptake of InVNBL kept increasing till 48 h p.i. along with an increasing tumor-to-blood ratio, and then declined gradually. The critical organs were those rich in reticularendothelial system, e.g. liver, spleen and small intestine.

### Therapeutic efficacy evaluation of liposomal drugs in tumor-bearing mice

**Figure 3:**
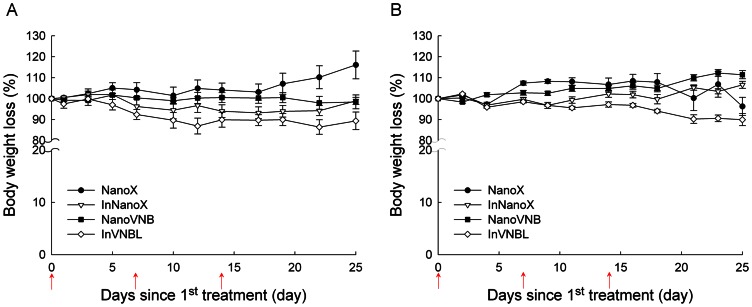
Body weight loss of the C26/tk-luc colon carcinoma-bearing mice after treatment with various liposomal drugs. The mice bearing large tumor (*n* = 9 for each group, A) and those bearing small tumor (*n* = 6 for each group, B) were injected intravenously with NanoX (•), InNanoX (▽), NanoVNB (▪) or InVNBL (◊) at 0, 7, and 14 days after first injection (arrow; three injections total). The zero time point indicates the initiation of therapy. Data were expressed as mean ± S.E.M.

NanoVNB, InNanoX, InVNBL and NanoX (as control) were individually administered into the tail veins of tumor-bearing mice at Day 0, 7, and 14 when the tumor size reached 58.4±8.0 mm^3^ (small-tumor groups) and 102.4±22.0 mm^3^ (large-tumor groups). In both small-tumor and large-tumor groups, maximal body weight loss was observed in InVNBL-treated mice at 25 days post the 1^st^ treatment (Fig. 3). In large-tumor group, the mean body weight loss in InVNBL-treated mice was 10.6%, while that in InNanoX- and NanoVNB-treated mice was only 1.1 and 1.7%. In small-tumor group, InVNBL-treated mice also lost 10.1% of body weights, while those treated with InNanoX and NanoVNB gained an average of 6.6 and 11.4% of body weights. The body weight changes in our treatment studies were significantly less than 20%, meet the general requirement of the drug treatment protocol [18].

**Figure 4:**
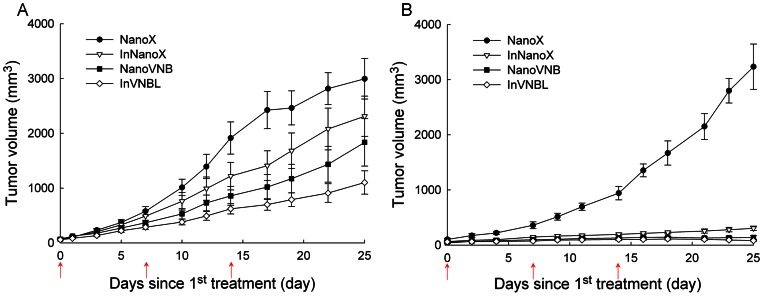
Tumor growth curves of the C26/tk-luc colon carcinoma-bearing mice after treatment with various liposomal drugs. The mice bearing large tumor (*n* = 9 for each group, tumor volume 102.4±22.0 mm^3^, A) and those bearing small tumor (*n* = 6 for each group, tumor volume 58.4±8.0 mm^3^, B) were injected intravenously with NanoX (•), InNanoX (▽), NanoVNB (▪) or InVNBL (◊) at 0, 7, and 14 days after first injection (arrow; three injections total). The zero time point indicates the initiation of therapy. Points, mean tumor sizes; bars, S.E.M.

Tumor growth was monitored by calipers measuring (thrice per week) and bioluminescence imaging (only for large-tumor groups) at designated time points till Day 25 since the 1^st^ treatment. For the mice bearing large tumors (Fig. 4A), combination treatment (InVNBL) achieved the maximum tumor growth inhibition (tumor size = 1102±213.1 mm^3^, *p*<0.01), followed by the chemotherapy (NanoVNB, tumor size = 1835±432.0 mm^3^, *p*<0.05), and then the radionuclide therapy (InNanoX, tumor size = 2312±366.9 mm^3^, *p*>0.05), compared with that of the control (NanoX, tumor size = 2996±370.6 mm^3^) at 25 days post 1^st^ treatment. The mean tumor growth inhibition rate (MGI) of InVNBL-, NanoVNB- and InNanoX-treated mice was 0.356, 0.604 and 0.762, respectively (Table 1). For the mice bearing small tumor (Fig. 4B), the most significant tumor growth suppression was observed in the combination treatment (InVNBL, tumor size = 80.4±9.58 mm^3^, *p*<0.01), followed by the chemotherapy (NanoVNB, tumor size = 138±33.9 mm^3^, *p*<0.01) and then the radionuclide therapy (InNanoX, tumor size = 304±39.6 mm^3^, *p*<0.01), compared with that of the control (NanoX, tumor size = 3235±411.2 mm^3^) at 25 days post 1^st^ treatment. The mean tumor growth inhibition rate (MGI) of InVNBL-, NanoVNB- and InNanoX-treated group was 0.007, 0.031 and 0.076, respectively (Table 1). Compared with those of the large-tumor group, the mice of small-tumor group displayed significantly superior tumor growth inhibition after treatment with InVNBL, NanoVNB and InNanoX (*p* = 0.001, 0.008 and 0.001, respectively). Synergistic tumor growth inhibition effect was demonstrated by the combination treatment (InVNBL) in the mice bearing large tumors (CI = 1.29, Table 1). For the mice bearing small tumors, even those treated with radionuclide-alone or chemodrug-alone liposomal drugs showed remarkable tumor growth inhibition, synergistic effect could not be observed in the combination treatment.

**Table 1:** The mean tumor growth inhibition rate of C26/tk-luc colon carcinoma-bearing mice on Day 25 since the 1^st^ treatment.

Liposomal drugs	Tumor growth inhibition		
	MGI	Expected^a^	CI^b^
**Large-tumor group**			
NanoX			
NanoVNB	0.604		
InNanoX	0.762		
InVNBL	0.356	0.460	1.29^c^
**Small-tumor group**			
NanoX			
NanoVNB	0.031		
InNanoX	0.076		
InVNBL	0.007	0.002	0.34

a Expected growth inhibition rate = growth inhibition rate of NanoVNB×growth inhibition rate of InNanoX.

b Combination index (CI) = expected growth inhibition rate/observed growth inhibition rate.

c Combination index larger than 1 indicates a synergistic effect, while that smaller than 1 indicates less than an additive effect.

**Figure 5:**
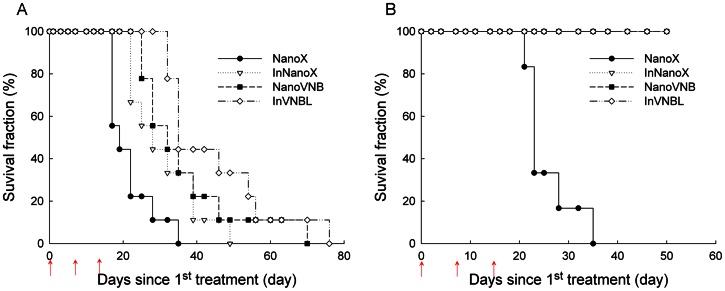
Survival fraction of the C26/tk-luc colon carcinoma-bearing mice after treatment with various liposomal drugs. The mice bearing large tumor (*n* = 9 for each group, A) and those bearing small tumor (*n* = 6 for each group, B) were injected intravenously with NanoX (•), InNanoX (▽), NanoVNB (▪) or InVNBL (◊) at 0, 7, and 14 days after first injection (arrow; three injections total). Mice were euthanized when tumor volume greater than 2500 mm^3^.

The survival fractions of mice treated with various PEGylated liposomal drugs were presented in Fig. 5. The mice bearing small tumor lived longer than those bearing large tumor in all kinds of treatment regimes. Consistent with the results observed in tumor growth inhibition study, InVNBL-treated mice owned the highest survival fraction compared to those treated with NanoVNB or InNanoX in the large-tumor groups. The mean survival time (MST) and median survival time of drug-treated mice were summarized in Table 2. For the large-tumor group, the MST of those treated with InVNBL (44.6±5.00 days, *p*<0.01), NanoVNB (36.4±4.79 days, *p*<0.05) and InNanoX (30.9±3.21 days, *p*<0.05) were significantly longer compared with the control mice (NanoX, 21.6±2.08 days). For the small-tumor group, the MST of mice treated with various regimes (InVNBL, NanoVNB and InNanoX) were all >50 days (till the end of the survival study) except the control group (NanoX, 25.5±2.33 days). The results clearly indicated that tumor burden is critical in cancer therapy when treated with PEGylated liposomal drugs.

**Table 2:** Survival time of C26/tk-luc colon carcinoma-bearing mice that treated with various liposomal drugs.

Liposomal drugs	Mean survival time ± S.E.M. (day)	Median survival time (day)
**Large-tumor group**		
NanoX	21.6±2.08	19
NanoVNB	36.4±4.79*	32
InNanoX	30.9±3.21*	28
InVNBL	44.6±5.00**	35
**Small-tumor group**		
NanoX	25.5±2.33	23
NanoVNB	>50**	>50
InNanoX	>50**	>50
InVNBL	>50**	>50

* : *p*<0.05,

** : *p*<0.01 compared with control group (NanoX).

### Monitoring tumor growth by bioluminescence imaging (BLI)

**Figure 6:**
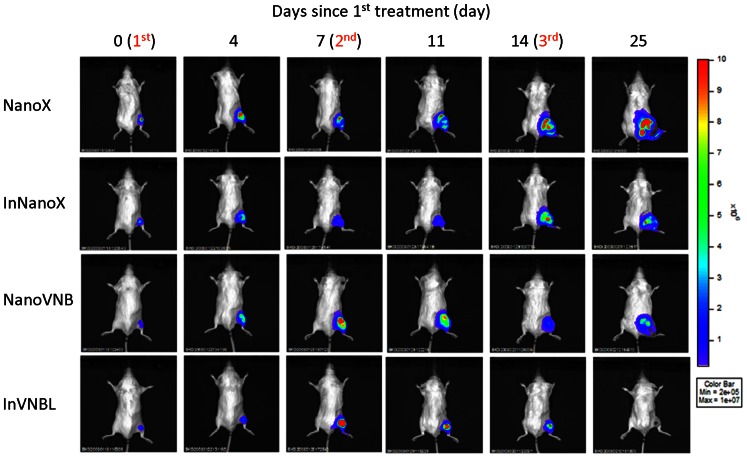
*In vivo* BLI of the C26/tk-luc colon carcinoma-bearing mice after treatment with various liposomal drugs. The large-tumor mice receiving various liposomal drugs were intraperitoneally injected with 150 mg/kg d-luciferin 15 min prior to image acquisition at designated time points. The photons emitted from the mice (positioned prone) were acquired for 1 minute. The mice were anesthetized with 1∼3% isoflurane while conducting imaging.

The tumor growth in large-tumor-bearing mice after liposomal drug treatment was sequentially monitored by BLI for a period of 25 days since the initiation of treatment (Fig. 6). The photon flux of tumor ROIs derived from bioluminescence images of mice all increased during the first 4 days post 1^st^ treatment, corresponding to the increasing tumor burden of mice in the four treatment groups (Fig. 4A). Subsequently, during the period of treatment till Day 25, the increment of tumor photon flux in InVNBL-treated mice became significantly less compared with those treated with NanoVNB and InNanoX, while that of the control mice increased rapidly in response to the fast growing tumors. The results of BLI revealed most dramatic tumor growth inhibition in the mice treated with InVNBL.

### Scintigraphic imaging

**Figure 7:**
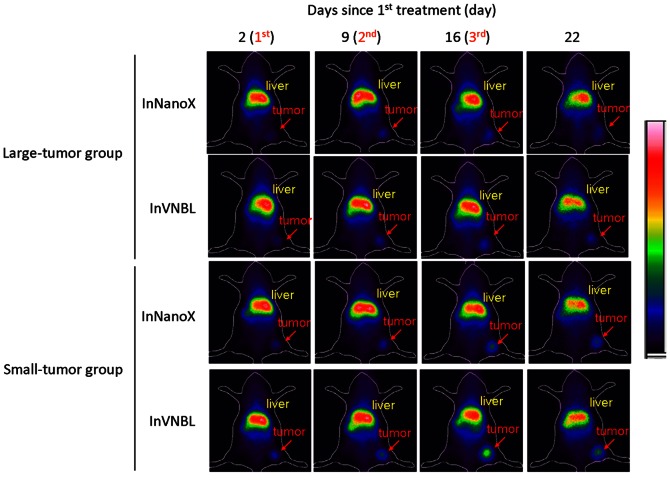
Whole-body scintigraphic images of the C26/tk-luc colon carcinoma-bearing mice at designated time points during the period of treatment with InNanoX and InVNBL. The scintigraphic imaging was performed for 20 min at 48 h after drugs administration (37 MBq/100 µL per injection) and at 8 days after the last course of treatment. The mice were anesthetized with 1∼3% isoflurane for all imaging. Tumor nodules are indicated by red arrows.

Whole-body scintigraphic imaging was conducted at 2 days post each time of treatment and 8 days after the third injection in mice that treated with ^111^In-containing liposomal drugs, InNanoX and InVNBL (Fig. 7). Significant radioactivity accumulation in tumor and liver was observed. The tumor accumulation kept increasing after each treatment (one injection per week, total three doses; Table 3). The specific tumor uptake (expressed in counts/pixel) was 28.00±4.75, 47.18±3.68 and 54.54±12.23 for InNanoX-treated mice; 34.20±5.05, 55.94±2.89 and 73.90±15.06 for InVNBL-treated mice in the large-tumor group at 2 days post each treatment. For the small-tumor group, the specific tumor uptake and the increment post each treatment were even higher (Table 3). The tumor-to-muscle ratio (T/M) reached 5.53±2.44 for InNanoX-treated mice and 9.60±2.58 for InVNBL-treated mice after the 3^rd^ treatment, higher than those observed in the large-tumor group (3.63±1.50 and 3.93±1.18, respectively).

**Table 3:** Estimated parameters derived from scintigraphic images of C26/tk-luc colon carcinoma-bearing mice acquired after intravenous injection of ^111^In-containing liposomal drugs InNanoX and InVNBL (37 MBq/100 µL).

Image acquisition date since the 1^st^ treatment	InNanoX			InVNBL		
	Counts/pixel^a^	T/M^a^	Volume (mm^3^)^b^	Counts/pixel^a^	T/M^a^	Volume (mm^3^)^b^
**Large-tumor group**						
2 (1 dose)	28.00±4.75	2.10±0.36	204.5±48.33	34.20±5.05	2.12±0.60	135.3±22.65
9 (2 doses)	47.18±3.68	3.22±0.77	759.6±160.0	55.94±2.89	3.54±0.58	381.5±52.75
16 (3 doses)	54.54±12.23	3.63±1.50	1409.0±270.8	73.90±15.06	3.93±1.18	700.0±104.7
22 (3 doses)	11.15±3.09	2.98±0.51	2079.0±381.5	13.58±5.13	3.01±1.25	909.4±171.0
**Small-tumor group**						
2 (1 dose)	38.66±7.14	2.67±0.69	86.43±4.2	46.36±10.58	3.12±0.64	62.29±7.32
9 (2 doses)	68.50±20.91	3.89±1.34	165.6±26.22	77.00±25.62	4.88±1.35	90.58±13.25
16 (3 doses)	95.34±29.10	5.53±2.44	213.5±26.2	114.2±27.06	9.60±2.58	102.5±15.79
22 (3 doses)	18.70±7.76	3.38±1.09	254.7±36.82	24.70±11.21	7.81±2.90	103.3±15.61

a Gamma photon counts per pixel (Counts/pixel) and tumor-to-muscle ratio (T/M) were determined from the ROI of tumor and muscle in the scintigraphic images.

b Tumor size (Volume, mean ± S.E.M.) was obtained by calipers measurement.

## Discussion

This study demonstrated passive targeting and selective accumulation in tumor after injection of liposomal drugs in a C26/tk-luc tumor xenograft model. An inverse profile between specific tumor uptake of InVNBL and tumor mass observed in this study echoed the previous reports. Harrington *et al.* have reported that when tumor burden were <0.1 g, 0.1–1.0 g and >1.0 g, the specific liposome uptake were 15.1±10.8, 5.9±2.2 and 3.0±1.3%ID/g, respectively [27]. The high level of liposome uptake in smaller tumors was caused by their relatively higher vascular volumes comprising immature, leaky neovasculature. The tumor uptake after injection of other macromolecular conjugates, like EGF and VEGF, also showed similar trend in different tumor size [20], [28]. Except tumor, high radioactivity accumulation in organs riched in reticular endothelial system, like liver and spleen, were noticed (data not shown). However, we have previously demonstrated that administration of InVNBL resulted in acceptable toxicity from the histopathologic studies and hematology analyses [18], [19] in animal studies.

Gutmann *et al.* has reported that head and neck tumor interstitial fluid pressure increased significantly with tumor size and showed good correlation with tumor volume [29]. Hilmas *et al.* demonstrated that both mean vascular surface area and vessel length per mm^3^ of tumor declined rapidly from 35 to 100 mm^3^ in tumor size, and the necrotic area increased from less than 5% in tumors of 35 mm^3^ to greater than 40% in tumors over 1500 mm^3^ [30]. For the small-tumor group (58.4±8.0 mm^3^) in this study, significant tumor growth inhibition was achieved in all three treatment regimes: combination treatment (InVNBL, MGI = 0.007), chemodrug treatment (NanoVNB, MGI = 0.031) and radionuclide therapy (InNanoX, MGI = 0.076). The lower interstitial fluid pressure, more microvasculature and less necrotic volume in the smaller tumor may enhance extravasation of liposomal drugs, and thus the synergistic effect of combination regimen cannot be observed (CI = 0.16). For the large-tumor group (102.4±22.0 mm^3^), a synergistic tumor growth inhibition was demonstrated by combination treatment (InVNBL, CI = 1.29). Treating with ^111^In- or VNB-encapsulated liposomal drug only resulted in limited tumor growth inhibition (InNanoX, MGI = 0.762; NanoVNB, MGI = 0.604). The poor therapeutic efficacy of InNanoX might due to a nonuniform drug distribution in the larger tumor. The short range of Augur electrons emitted from In-111 could not effectively damage the tumor cells in distance. The tumor microenvironment is tumor size related, would influence the accumulation and microdistribution of PEGylated liposomal drugs in tumor, and contribute to the therapeutic efficacy. In all, after treating the tumor-bearing mice with NanoVNB, InNanoX and InVNBL, the optimal tumor control and highest survival rate were achieved by combination therapy, followed by chemotherapy and then radionuclide therapy (Fig. 4, 5 and Tables 1, 2).

Combination regimen is an advanced treatment strategy for cancer therapy. It is assumed that we can improve the treatment efficacy of various therapeutic modalities to have synergistic anticancer effects with reduced toxicity or side effects [31]. The strategy of combined radionuclide- and chemo-therapy has achieved better tumor treatment efficacy and lower toxicity to normal tissues [32]. Our recent approach is using PEGylated liposomes to carry both radionuclide and chemodrug [24], [33]. Chen, Behr, Howell and Mariani *et al.* have demonstrated that Auger electrons-emitters (e.g. In-111 and I-125) may exhibit biological effects and antitumor efficacy similar to the typical high-LET radiation, such as α-emitters, assumed that the Auger electrons-emitters have internalized into the cells, or even the cell nuclei [20], [34], [35], [36]. Indeed, Auger electron-emitters decaying in the neighborhood of DNA produce a significant amount of chemically reactive radical species (e.g. OH·, H·, e^−^_(aq)_ etc.), which can generate DNA double-strand breaks. Edelstein *et al.* have shown that vinorelbine can potentiate the antitumor effect of radiation and is cell cycle dependent [37]. The maximal effect is achieved when the cells are in the G2/M-phase. Terasima and Sinclair *et al.* have demonstrated that cells in the late G2/M-phase are more radiosensitive than in other phases [38], [39]. Fukuoka *et al.* have reported that vinorelbine, even at a minimally toxic concentration, could sensitize human NSCLC cells to external radiation moderately [40]. In our study, vinorelbine, besides its cytotoxic effect, may serve as a radiosensitizer in the colon carcinoma tumor cells by arresting them in G2/M-phase. The G2/M-arrested tumor cells would become more susceptible to induce apoptosis by radiations from the radionuclide, In-111.

Noninvasive molecular imaging such as positron emission tomography, single photon emission computed tomography, magnetic resonance imaging and optical imaging have gradually expanded to the drug discovery and development in preclinical studies [41], [42]. In this study, tumor growth inhibition and tumor uptake of ^111^In-labeling liposomal drugs in the tumor-bearing mice during the period of therapy were monitored by bioluminescence and scintigraphic imaging (Fig. 6, 7). Bioluminescence imaging of large-tumor mice revealed significant tumor growth suppression during the period of combination treatment with InVNBL. The noninvasive *in vivo* optical imaging is a useful technique for evaluation of therapeutic response in real-time and longitudinal monitoring. In addition, both primary tumors and micrometastases could be detected by BLI *in vivo* [43], [44]. It could provide sensitive detection and quantitative assay of tumor growth and metastasis for our future investigation of tumor treatment in a colon carcinoma/ascites-bearing mouse model. We have demonstrated that i.p. injection of InVNBL in the tumor/ascites-bearing mouse model is a promising treatment approach for peritoneal malignancies [22]. The small lesion of tumor, e.g. at the early stage of tumor metastases, would also be suitable for liposomal drugs treatment [33], [45].

Drug accumulation in tumor is a major predictor of tumor response for cancer therapy in tumor mouse models [46]. Tumor uptake of ^111^In-encapsulated liposomal drugs in mice can be clearly visualized by scintigraphic imaging. Intense accumulation of InNanoX/InVNBL in tumor was observed 2 days post injection, and persisted in the tumor region till 8 days after the 3^rd^ course of treatment (Fig. 7). Abu *et al.* reported on 2012 that two sequential injections of PEG-coated liposomal drug resulted in higher apoptotic activity and broader intratumor distribution in tumor tissue than single injection [47]. In the present study, significantly increased drugs accumulation in tumor after periodical treatment with ^111^In-labeled liposomal drugs was observed by noninvasive scintigraphic imaging. The stepwise increased tumor uptake may be attributed, at least in part, to the changes in tumor microenvironment during therapy. Furthermore, the treated mice that revealed the most significant tumor growth inhibition (InVNBL-treated mice in the small-tumor group) were also the mice that showed the highest tumor uptake and T/M ratio in scintigraphic imaging. The scintigraphic imaging post administration of theranostic radionuclide-encapsulated liposomes, such as InNanoX or InVNBL in the present study, would be a potent noninvasive tool to screen patients before conducting tumor treatment with liposomal drugs.

## Conclusion

Tumor burden is a critical, and even determining, factor in cancer therapy using PEGylated liposomal drugs. Mice bearing smaller tumor exhibits higher specific tumor uptake, and higher tumor uptake of drugs results in more significant tumor growth inhibition. Initiating tumor treatment when it is relatively small or in the early stage of metastasis would be most efficatious. The scintigraphic imaging of ^111^In-encapsulated liposomes would provide a noninvasive screening of patients before conducting tumor treatment. The liposomal drugs labeled with ^111^In could fulfill both diagnostic and therapeutic purposes.
